# Novel aspects of Sjögren’s syndrome in 2012

**DOI:** 10.1186/1741-7015-11-93

**Published:** 2013-04-04

**Authors:** Angela Tincani, Laura Andreoli, Ilaria Cavazzana, Andrea Doria, Marta Favero, Maria-Giulia Fenini, Franco Franceschini, Andrea Lojacono, Giuseppe Nascimbeni, Amerigo Santoro, Francesco Semeraro, Paola Toniati, Yehuda Shoenfeld

**Affiliations:** 1 Rheumatology and Clinical Immunology Unit, Spedali Civili, Piazzale Spedali Civili 1, 25100 Brescia, Italy; 2 Chair of Rheumatology, Department of Clinical and Experimental Sciences, University of Brescia, Spedali Civili, Piazzale Spedali Civili 1, 25100 Brescia, Italy; 3 Rheumatology Unit, Department of Medicine, University of Padua, Azienda Ospedaliera di Padova, Via Giustiniani 2, Padua, 35128, Italy; 4 Rheumatology Unit, Vallecamonica Hospital, Via Manzoni 142, Esine (Brescia), 25040, Italy; 5 Obstetrics and Gynecology Unit, Spedali Civili and University of Brescia, Piazzale Spedali Civili 1, Brescia, 25100, Italy; 6 Ophthalmology Unit, Spedali Civili and University of Brescia, Piazzale Spedali Civili 1, Brescia, 25100, Italy; 7 Pathology Unit, Spedali Civili, Piazzale Spedali Civili 1, Brescia, 25100, Italy; 8 Zabludowicz Center for Autoimmune Diseases, Sheba Medical Center, Sackler Faculty of Medicine, Tel-Aviv University, Tel Hashomer, 52621, Israel

**Keywords:** Anti-B cell therapies, Autoantibodies, Autoimmune diseases, Chronic sialoadenitis, Dry eye syndrome, Lymphoma, Peripheral neuropathy, Sicca syndrome, Sjögren’s syndrome, Vitamin D

## Abstract

Sjögren’s syndrome (SS) is a systemic progressive autoimmune disease characterized by a complex pathogenesis requiring a predisposing genetic background and involving immune cell activation and autoantibody production. The immune response is directed to the exocrine glands, causing the typical ‘sicca syndrome’, but major organ involvement is also often seen. The etiology of the disease is unknown. Infections could play a pivotal role: compared to normal subjects, patients with SS displayed higher titers of anti-Epstein-Barr virus (EBV) early antigens, but lower titers of other infectious agent antibodies such as rubella and cytomegalovirus (CMV) suggest that some infections may have a protective role against the development of autoimmune disease. Recent findings seem to show that low vitamin D levels in patients with SS could be associated with severe complications such as lymphoma and peripheral neuropathy. This could open new insights into the disease etiology. The current treatments for SS range from symptomatic therapies to systemic immunosuppressive drugs, especially B cell-targeted drugs in cases of organ involvement. Vitamin D supplementation may be an additional tool for optimization of SS treatment.

## Introduction

Sjögren syndrome (SS) is a chronic autoimmune inflammatory disease that primarily involves the exocrine glands, resulting in their functional impairment. The syndrome can present either alone (primary Sjögren’s syndrome (pSS)) or in the context of an underlying connective tissue disease, most commonly rheumatoid arthritis (RA) or systemic lupus erythematosus (SLE) (secondary Sjögren’s syndrome (sSS)) [1].

SS is the second most common autoimmune rheumatic disease, with an estimated prevalence ranging from 0.1 to 4.8% in different studies. It mainly affects middle-aged women, with a female to male ratio reaching 9:1 [2].

Although the etiology of SS remains unknown, susceptibility to the disease can be ascribed to the interplay between genetic, environmental and hormonal factors. The chronic immune system stimulation is thought to play a central role in the pathogenesis of the disorder, as illustrated by several indices of immunological hyperactivity, including various autoantibodies, in particular anti-Ro/SS-A (anti-Ro) and anti-La/SS-B (anti-La) [1].

A genetic predisposition to SS has been suggested [3]. Familial clustering of different autoimmune diseases and coassociation of multiple autoimmune diseases in individuals have both frequently been reported. It is common for a SS patient to have relatives with other autoimmune diseases (30%) [1]. The polymorphic major histocompatibility complex (MHC) genes are the best documented genetic risk factors for the development of autoimmune diseases; with regard to SS, DRB1*0301-DQB1*0201-DQA1*0501 haplotypes are the strongest risk factors for the formation of an anti-Ro/La response and to the development of the disease [4].

Although many human leukocyte antigen (HLA) haplotypes have been found in SS subjects from different ethnic boundaries, the majority of patients with SS carry a common allele, DQA1*0501, probably involved in predisposition to the disease [5]. Regardless, no significant different geographic distribution has been described in pSS to date [6].

In the current review we present the complexity of SS from different points of view, reporting on the current cutting edge of knowledge about this disease. This multidisciplinary approach to SS is the result of a symposium held in Brescia (Italy) in June 2012, involving several specialists who take care of different aspects of the diagnosis, management and therapy of SS.

### Clinical features and classification

SS typically presents as dry eyes (xerophthalmia or keratoconjunctivitis sicca (KCS)) and dry mouth (xerostomia) [7]. KCS usually presents insidiously over a period of several years. Affected patients may describe a ‘gritty’ or ‘sandy’ feeling in their eyes [8]. Complications of xerophthalmia include corneal ulceration and infection of the eyelids. Dryness of the mouth may give rise to difficulties in the swallowing of dry foods without fluid, and need for frequent small sips of water, also at night. Loss of the protective and antimicrobial properties of saliva may increase dental caries and predispose patients to oral candidiasis. Parotid swelling and other xeroses, such as dryness of the nose, throat, skin, and vagina, also often occur [8].

Establishing the diagnosis of SS is often difficult. The symptoms are non-specific: sicca symptoms are extremely common, especially in older patients, partly due to age-related atrophy of secreting tissues and partly due to other conditions, especially use of drugs.

No single laboratory test allows for definitive diagnosis of SS. However, a combination of abnormal test results is frequently observed: elevated erythrocyte sedimentation rate (ESR), mild normochromic normocytic anemia, leukopenia and polyclonal hypergammaglobulinemia.

Autoantibodies are present in the majority of SS cases: rheumatoid factor (RF), Anti-nuclear antibodies (ANA) and anti-Ro and anti-La are strongly indicative of SS, although not exclusive [8].

There is no single disease-specific diagnostic criterion for SS. The most widely used classification criteria are those revised in 2002 by a joint effort by research groups in Europe and in the USA (American-European Consensus Group (AECG)) (Table 1) [9,10]. In addition to the subjective symptoms of dry eyes and dry mouth, the following objective signs should be present: ocular signs by Schirmer’s I test and/or Rose Bengal score; focal sialadenitis by histopathology; salivary gland involvement by salivary scintigraphy, parotid sialography or unstimulated salivary flow; and autoantibodies of anti-Ro and/or anti-La specificity. The diagnostic role of the histopathology of minor salivary glands has been considered important and is currently considered as a ‘gold standard’, although a recent meta-analysis has shown that the diagnostic usefulness has actually only been evaluated in a few studies [11].

**Table 1:** Comparisons between the 2002 and 2012 criteria for Sjögren’s syndrome (SS)

**2002 criteria from the American-European Consensus Group (AECG) (Vitali *et al.***[9]**)**	**2012 criteria from the Sjögren’s International Collaborative Clinical Alliance (SICCA) (Shiboski *et al*.**[12]**)**
(I) Ocular symptoms (positive response to at least one of three):	(I) Ocular symptoms:
Daily, persistent, troublesome eyes for more than 3 months	Not included
Recurrent sensation of sand or gravel in the eyes	
Use of tear substitutes more than three times per day	
(II) Oral symptoms (positive response to at least one of three):	(II) Oral symptoms:
Daily feeling of dry mouth for more than 3 months	Not included
Recurrently or persistently swollen salivary glands as an adult	
Frequent drinking of liquids to aid in swallowing food	
(III) Ocular signs (positive result for at least one of two tests):	(III) Ocular signs:
Schirmer’s test, performed without anesthesia (≤5 mm in 5 minutes)	Keratoconjunctivitis sicca with ocular staining score ≥3, according to Whitcher *et al*. [13] (preferential use of fluorescein staining or lissamine green staining, but break-up time; and unanesthetized Schirmer’s test can also be used). It is assumed that individual is not currently using daily eye drops for glaucoma and has not had corneal surgery or cosmetic eyelid surgery in the last 5 years).
Rose Bengal score or other ocular dye score (≤4 according to van Bijsterveld’s scoring system)	
(IV) Histopathology in minor salivary gland biopsy:	(IV) Histopathology in minor salivary gland biopsy:
Focal lymphocytic sialoadenitis, with focus score ≥1 (a focus is defined as ≥50 lymphocytes per 4 mm^2 ^of glandular tissue adjacent to normal appearing mucous acini)	Focal lymphocytic sialadenitis, with a focus score ≥1 (a focus is defined as ≥50 lymphocytes per 4 mm^2 ^of glandular tissue adjacent to normal appearing mucous acini)
(V) Salivary gland involvement (positive result for at least one of three):	(V) Salivary gland involvement:
Unstimulated whole salivary flow (≤1.5 ml/15 minutes)	Not included
Parotid sialography showing the presence of diffuse sialectasis (punctuate, cavitary or destructive pattern), without evidence of obstruction in the major ducts	
Salivary scintigraphy showing delayed uptake, reduced concentration and/or delayed excretion of tracer	
(VI) Autoantibodies:	(VI) Autoantibodies:
Presence in the serum of antibodies to Ro (SS-A) or La (SS-B) antigens, or both	Positive serum anti-SS-A/Ro and/or anti-SS-B/La or (positive rheumatoid factor and anti-nuclear antibody (ANA) titer ≥1:320)
Classification criteria:	Classification criteria:
Primary SS:	At least two of the three items in order to classify a patient as SS
The presence of any four of the six items, as long as either item IV (histopathology) or item VI (serology) is positive	
The presence of any three of the four objective criteria (items III, IV, V and VI)	
Secondary SS:	
In the presence of another connective tissue disease, the presence of item I or item II, plus any two from items III, IV and V	
Exclusion criteria:	Exclusion criteria:
Past head and neck radiation treatment, hepatitis C infection, AIDS, pre-existing lymphoma, sarcoidosis, graft versus host disease, use of anticholinergic drugs (since a time shorter than fourfold the half-life of the drug)	History of head and neck radiation treatment, hepatitis C infection, AIDS, sarcoidosis, amyloidosis, graft versus host disease, IgG4-related disease

Recently, the Sjögren’s International Collaborative Clinical Alliance (SICCA) proposed a new expert consensus approach consisting of classification criteria based entirely on objective measures [12]. In particular, not only have ocular and oral symptoms have been deleted, but also the study of salivary gland involvement has been excluded from the criteria (Table 1).

In fact, the evaluation of salivary gland involvement in SS is still a matter of debate. In addition to standard tests for assessment of salivary gland involvement, namely the unstimulated salivary flow test, salivary gland scintigraphy and contrast sialography, other methods have been studied such as magnetic resonance sialography and ultrasonography (US) [14]. It has been suggested that US may provide useful diagnostic information comparable to that of biopsy of the minor salivary glands, but US is less expensive and non-invasive [15,16].

### Differential diagnosis of ‘dry eye’

The importance of objective tests for the definition of ocular dryness has been stressed by the 2012 criteria [12]. Therefore, correct evaluation of a ‘dry eye’ becomes critical in the investigation of patients with a suspected case of SS.

Dry eye syndrome is a common but very complex disorder of the tear film. Over the last few decades substantial progress has been made in understanding the structural elements of the tear film and ocular surface leading ultimately to revised concepts about the way in which the tear film is formed and maintained, and the pathophysiologic events operative in the development of dry eye.

**Figure 1:**
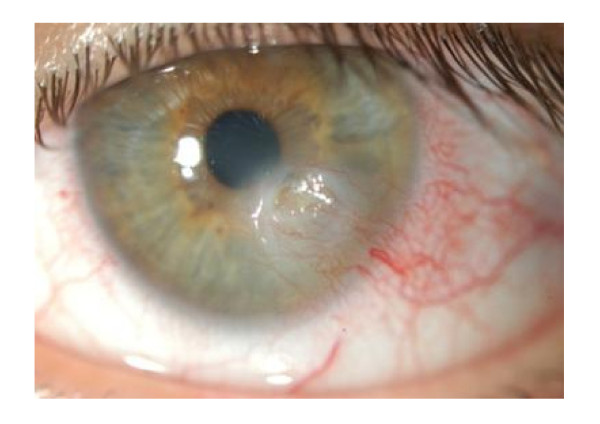
Severe ocular surface damage in a dry eye patient.

The structure of tear film can be subdivided into an anterior lipid layer, a middle aqueous layer and an innermost mucin layer. Meibomian glands, lacrimal glands, goblet cells and epithelial cells of the ocular surface produce these layers. In the 1980s, for the first time, researchers started to consider that the ocular surface is a functional unit (lacrimal functional unit (LFU)), and its components are represented by the lacrimal gland, corneal epithelium, conjunctival epithelium and goblet cells, tear film and the eyelid border with Meibomian glands [17]. The ocular surface is essential for visual function and is considered as an interface between the external environment and the host. Hydrodynamic factors, such as eyelid blinking and closure, are essential to maintain ocular surface functionality. In 1995, the Dry Eye Study Group [18] described ‘dry eye syndrome’ as a tear film pathology that occurs due to either decreased tear production or increased evaporation. It causes damage to the interpalpebral ocular surface and is associated with a variety of symptoms reflecting ocular discomfort (Figure 1). Until recently this was the common definition of ‘dry eye syndrome’. However, the international report of the Dry Eye Workshop (DEWS) changed the definition of dry eye in 2007 [19]. According to DEWS, ‘dry eye syndrome’ is a multifactorial disease of the tears and ocular surface that results in symptoms of discomfort, visual disturbance, and tear film instability with potential damage to the ocular surface itself. It is accompanied by increased osmolarity of the tear film and inflammation of the ocular surface. The tear film is a very important and highly dynamic part of the ocular surface system, which promptly responds to pathological events with modifications of quantity and quality of tear production, an increase in proliferation and migration of epithelial cells and permeability of conjunctival vessels. Another crucial component of the system is the neural network. Sensory receptors monitor conditions of the tears and cells, sending afferent signals to the central nervous system that, in turn, send efferent impulses mainly to the secretory glands and cells, effecting changes in composition and volume to maintain homeostasis and to respond to injury and stress. As stated before, dry eye is a multifactorial disorder of the LFU; it involves several interacting mechanisms. Dysfunction of any component can lead to dry eye disease by causing alterations in the volume, composition, distribution, stability and clearance of the tear film. A key role is played by decreased tear production and epithelial damage along with tear hyperosmolarity and tear film instability. Altogether these events start self-perpetuating and mutually reinforcing complex global mechanisms, ultimately leading to ocular surface inflammation [20]. The latter, regardless of the initiating event, is a key factor perpetuating dry eye.

**Figure 2:**
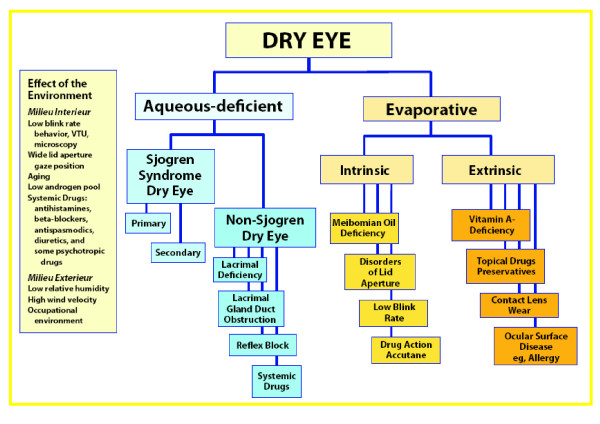
**Dry eye classification flow chart (Dry Eye Workshop Report, 2007) **[19]**.** The causes of ‘dry eye syndrome’ are subdivided into aqueous-deficient and evaporative groupings. Sjögren’s syndrome (SS) belongs to the first group. Modified from [19].

According to the DEWS report, dry eye disease comprises two major etiopathogenic groups: evaporative dry eye and aqueous tear-deficient dry eye (Figure 2). Hyperevaporative dry eye can be due to intrinsic and extrinsic causes. Among the first group are changes in tear composition, eyelid disorders, incomplete blinking or reduced blinking rate, ocular surface irregularities and drug action. Extrinsic causes include, among others, vitamin A deficiency, topical drug preservatives, contact lens wear and ocular surface disease. Aqueous tear deficient dry eye can be subdivided into SS and non-SS syndrome dry eye groups. The latter group has several primary causes, including the lack of a lacrimal gland (congenital or acquired), impairment or dysfunction of the lacrimal gland, reflex block and drug action. Non-SS dry eye can also be secondary to a variety of conditions. SS dry eye is associated with autoimmune inflammation in the lacrimal glands [19].

It should be remembered, however, that instances of hyperevaporative and aqueous tear deficient dry eye in most cases are not clinically so well defined and often there is a certain degree of overlap between these two dry eye groups. A hyperevaporative dry eye over time also becomes aqueous tear deficient, and vice versa, making it difficult to precisely classify the condition.

**Figure 3:**
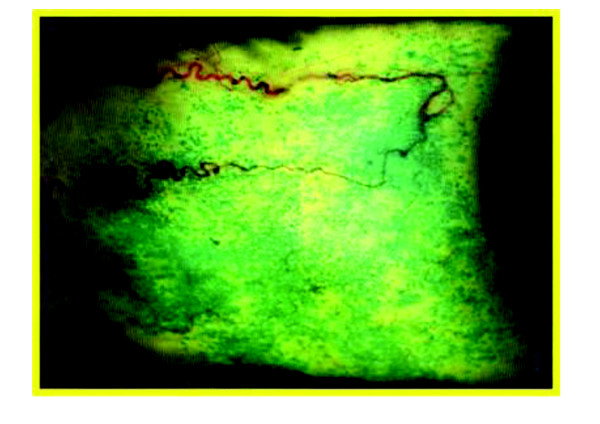
Lissamine green staining of the conjunctiva in dry eye syndrome.

Ophthalmologists should recognize and diagnose dry eye syndrome to prevent or treat ocular surface pathologies. As it may be associated with a variety of causes, it is important to perform a comprehensive evaluation. This should include a complete clinical history, accurate examination of the patient (skin, blinking rate, eye and lid morphology, and so on), a slit-lamp examination and laboratory tests that can help towards a diagnosis of dry eye related to SS. Lacking a single conclusive test to diagnose dry eye syndrome, a lot of different procedures, easy or complicated, cheap or expensive and more or less useful, have been developed in order to help ophthalmologists. The tests of choice to diagnose dry eye include break-up time (BUT), ocular surface staining and Schirmer’s test [12]. The reasons for their use lay in their reproducibility, sensitivity and in the fact that all of them are quite easy to perform. Ocular surface dyes used in clinical practice are fluorescein and lissamine green (Figure 3). Rose Bengal was progressively abandoned because of patient discomfort. The lissamine green dye test is very sensitive and stains damaged epithelial cells without causing any discomfort to patients.

Once a diagnosis is confirmed, management of dry eye depends on the cause and severity of the condition. New treatment approaches are designed to modify the underlying disease process. Every associated condition must be treated. Therapy should normalize the tear film, decrease ocular surface inflammation, stimulate epithelial healing, improve neural feedback, decrease lacrimal gland inflammation and improve its function.

In conclusion, therapy should be aimed at protecting the ocular surface, alleviating the signs and symptoms of dry eye and, most importantly, at breaking the vicious cycle leading to chronic inflammation, thus improving the quality of life of patients.

### Not only ‘sicca syndrome’: extraglandular manifestations of SS

Despite glandular involvement being the major and typical feature of pSS, this autoimmune disease can have several systemic manifestations. In fact, 30% to 70% of patients develop systemic involvement before or after the diagnosis of pSS [21-24]. In addition, it must be considered that this group of patients more commonly has circulating anti-Ro and anti-La autoantibodies, in comparison with the group of patients with sicca-limited disease [25].

Most extraglandular manifestations, similar to the exocrine gland involvement, can be considered as expression of the so-called ‘autoimmune epithelitis’ because the primary target of the autoimmune response is the epithelial component [26,27]. Nevertheless, in other clinical manifestations the pathogenesis seems to be completely different as it may involve vasculitis and/or immune complex deposition and complement activation, as is the case in skin vasculitis, glomerulonephritis and peripheral neuropathy.

One of the most frequent symptoms in pSS is represented by fatigue, prominent in approximately 70% of patients. Though the instruments for fatigue assessment are still inadequate or lacking, it seems that the physical and somatic rather than mental aspects of fatigue are more severely and frequently affected in pSS [28]. A moderate correlation between depression and fatigue has also been found. One possible explanation is that fatigue and depression share common underlying biological mechanisms.

Respiratory manifestations are frequently detected but they are clinically significant in only 10% of patients [29]. The more common clinical symptoms are represented by nasal crusting, epistaxis, recurrent sinusitis, dry cough and dyspnea. They are caused by dry nose, dry trachea, small airway obstruction and/or interstitial lung disease (ILD). Non-specific interstitial pneumonia (NSIP) seems to be the most frequent histological pattern of ILD but lymphocytic IP (LIP) and usual IP (UIP) are also present.

The kidneys are often involved in pSS. The major clinicopathological entity is interstitial nephritis (IN), which appears early or even may precede the onset of sicca symptoms [30]. Distal renal acidosis (both type I and II) is the most frequent clinical presentation. Aside from IN, glomerulonephritis (GMN) is more rarely detected in patients with pSS and is strongly associated with low C4 levels and mixed cryoglobulinemia. However, IN is frequently subclinical and overt clinical renal disease is detectable in approximately 5% of patients with pSS, in which IN and GMN are almost equally distributed.

With regard to skin involvement, nearly half of all patients with pSS may present cutaneous manifestations consisting of skin xerosis, angular cheilitis, erythema anulare, chilblain lupus and skin vasculitis that includes flat or palpable purpura and urticarial vasculitis [31].

Arthralgias are commonly reported in patients with pSS while typical non-erosive arthritis is less frequent [32]. Likewise, myalgias are frequent while myositis is rarely diagnosed in pSS.

Gastrointestinal manifestations include nausea, dysphagia or epigastric pains that are frequently due to dryness of the pharynx and esophagus or to esophageal dysmotility and gastritis. The typical histological pattern is chronic atrophic gastritis with lymphoid infiltration. Hyperamylasemia is rather frequent, though very rarely it is an expression of acute or chronic pancreatitis. Abnormal liver tests are not uncommon but autoimmune hepatitis is diagnosed in 1.7% to 4% of patients with pSS, while autoimmune cholangitis (with histological changes similar to stage I primary biliary cirrhosis) develops mainly within the 5% to 10% of patients with antimitochondrial antibodies [33].

Approximately 20% of patients with pSS develop autoimmune thyroiditis (primarily Hashimoto thyroiditis and to a lesser extent, Graves’ disease) and more than 50% of them have subclinical hypothyroidism. Autoantibodies against thyroid peroxidase (anti-TPO) and thyroglobulin (anti-TG) can be used as primary indicators of patients who are prone to developing thyroid disease in the future [34].

The prevalence of neurological manifestations in pSS varies between 2% to 60% with pure or predominantly sensory polyneuropathies being the most common manifestations (for example, sensory ataxic or small fiber sensory painful neuropathy) [21,35]. Sensorimotor polyneuropathy and polyradiculopathy, mononeuritis multiplex, autonomic neuropathy (for example, Adie’s pupils and orthostatic hypotension), trigeminal and other cranial neuropathies are other manifestations of the involvement of peripheral nervous system (PNS) in pSS. Central nervous system involvement is much less common than PNS involvement, with multiple sclerosis-like changes, seizures, transverse myelitis, aseptic meningitis, optic neuritis, diffuse encephalopathy and dementia as reported manifestations [36].

### Pathogenesis, histopathology and progression to lymphoma

The pathological hallmark of SS is a chronic inflammatory infiltrate in the exocrine glands, mainly constituted by activated T and B cells [37,38]. The immune-mediated damage appears in the apoptosis of glandular epithelial cells [39] and seems to be mediated by several proinflammatory T helper 1-type cytokines [40]. The epithelial cells of salivary glands from patients with SS also display alterations in cell adhesion and shape [41]. The immune dysregulation seems to be orchestrated by genetic factors, including certain HLA phenotypes and polymorphisms in genes encoding cytokines or factors implicated in cytokine signaling, by the environment (such as viruses) and by the hormonal milieu [42].

The histopathological picture of SS is the chronic periductal sialoadenitis [43]. In the early stages of disease, focal aggregates of lymphocytes appear in the glandular lobules. Initially, the lymphocytes infiltrate the space around small interlobular-intralobular ducts, and subsequently they determine the atrophic involution of the acina. The lymphocyte infiltrate then spreads from the periductal position to the parenchyma, with the final result of a diffuse infiltration of lymphocytes and loss of tissue architecture. In addition, the lymphocytes initiate the damage to the ducts with the formation of epimyoepithelial lesions. As a result, hyaline material, similar to a basal membrane, is present in the lumen of the ducts. Of note, some morphological alterations described in SS (‘epimyoepithelial sialoadenitis’) can also be found in the absence of overt disease (no clinical and serological features of SS). Such a histopathological picture can be defined as a ‘benign lymphoepithelial lesion’.

**Figure 4:**
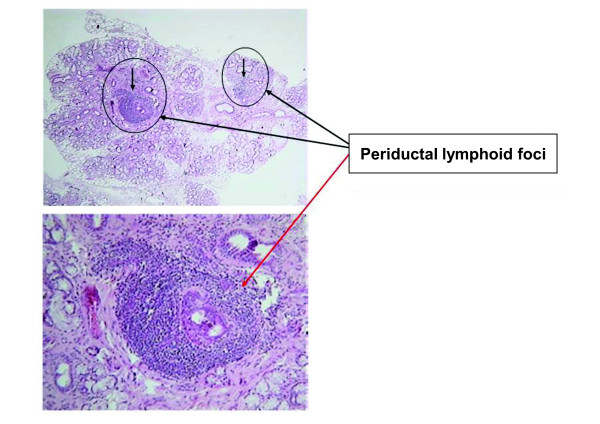
**Microscopy of a minor salivary gland in Sjögren’s syndrome (SS): ‘periductal lymphoid focus’. **The finding of at least 1 focus (periductal aggregate of at least 50 lymphocytes, mostly CD4+) in 4 mm^2 ^of tissue is diagnostic for SS. The ‘score’ is the number of foci in 4 mm^2^ of tissue.

According to the international guidelines [9,12], the histological criteria for the definition of SS are both qualitative and quantitative: the ‘focus’ must be composed of at least 50 lymphocytes infiltrating the periductal area; 1 focus must be detected in a tissue area of at least 4 mm^2^ (see Figure 4).

The main complication of SS is hematological neoplasia. Compared to healthy individuals, patients with SS have a 10 to 50 times higher risk of lymphoma and, according to a large case series, 2% to 9% of patients with SS develop lymphoma [7].

The parotid gland is affected in the majority of cases and the most frequent type of non-Hodgkin’s (NH) lymphoma is the marginal zone lymphoma of the mucosa-associated lymphoid tissue (MALT). Such lymphomas can also be found in other organs (stomach, lungs and kidney). Other types of lymphoma are rare in SS: Hodgkin’s lymphoma, B cell NH lymphoma with diffuse giant cells and centrofollicular histotypes and T cell NH lymphoma [44].

**Figure 5:**
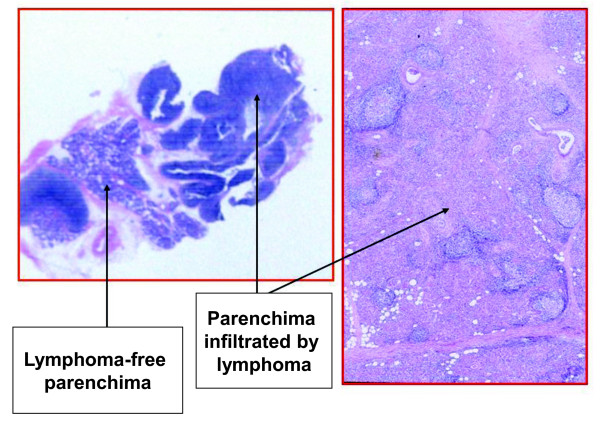
**Microscopy of non-Hodgkin’s (NH) marginal B cell lymphoma. **The most frequent lymphoma in Sjögren’s syndrome (SS) is the NH marginal B cell type, which comes from mucosa-associated lymphoid tissue (MALT).

**Figure 6:**
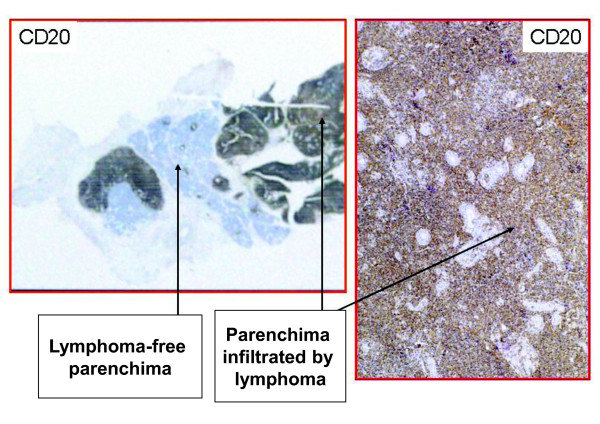
**Anti-CD20 staining of non-Hodgkin’s (NH) marginal B cell lymphoma. **The lymphoid infiltrate is constituted by medium-sized cells, with a cleaved nucleus and a large cytoplasm, which are factors strongly positive for CD20.

Despite the inflammatory infiltrate in the salivary glands being mostly made up of T cells, the development of lymphoma involves the B cells. The lymphoma cells in the marginal zone NH type are medium-sized cells with a cleaved nucleus and large cytoplasm (Figure 5) and with a positive CD20 reaction (Figure 6). Such cells cluster in the epimyoepithelial islets. Initially, there might be several different clones of B cells, but over time a single clone can progressively expand and invade the glandular parenchyma with the formation of a lymphoma.

Risk factors for the development of lymphoma have been identified in patients with SS and include the presence of palpable purpura, low C4 and mixed monoclonal cryoglobulinemia. Patients displaying these risk factors should be monitored closely [44].

### Serological profile

SS is an autoimmune disease characterized by a strong polyclonal B cell activation and different circulating autoantibodies, classically represented by anti-nuclear antibodies, anti-Ro, anti-La, rheumatoid factor and, more rarely, anti-centromere, anti-Ki/SL, anti-Ku or anti-p80 coilin.

Other autoantibodies have been described in SS, probably involved in the pathogenesis of different aspect of the disease, such as anti-α fodrin [45], anti-carbonic anhydrase, and anti-muscarinic receptor antibodies [46].

Anti-Ro and anti-La antibodies are considered the classical hallmark of SS, detected in about 60% and 40% of SS, respectively, and included in both the AECG and SICCA classification criteria [9,12]. Anti-Ro and anti-La antibodies define a disease with a higher rate of extraglandular manifestations and more active immunological status, when compared with ‘seronegative’ SS cases (without anti-Ro or anti-La antibodies). Anti-Ro/La-positive patients with SS can show severe hypergammaglobulinemia, cryoglobulins and a high risk of developing lymphoma [47].

Circulating levels of anti-Ro/La do not correlate with disease activity; regardless, the IgA anti-Ro titer seems to be associated with the rate of lymphocyte glandular infiltration. In addition, the expression of La and 60 kDa Ro antigens in ductal glandular cells could represent a trigger for inducing and maintaining a local inflammation and tissue-specific immune response. Moreover, a strong correlation was found between circulating autoantibodies and Ro/La producing cells in salivary glands [48]. Anti-Ro and anti-La autoantibodies from SS sera, but not healthy IgG, can activate caspase 3 and determine apoptosis in human salivary gland cells, *in vitro*[49]. In addition, anti-Ro/SS-A antibodies stimulate the production of proinflammatory cytokines interleukin (IL)-6 and IL-8 by human healthy salivary gland epithelial cells [50]. Therefore, these autoantibodies seem to have a pathogenic role for the impairment of the secretory function in the salivary glands.

Anti-Ro antibodies recognize a macromolecular complex, constituted by 60 kDa and 52 kDa Ro proteins and short cytoplasmic RNA molecules. The 60 kDa and 52 kDa Ro antigens are encoded by different genes and are completely different in amino-acid sequence, epitopes and biological role within the cell. The 60 kDa Ro is directly bound to RNA of the Ro complex and seems to have a role in DNA replication. By contrast, 52 kDa Ro does not contain an RNA-binding sequence but could be considered part of the ‘Ro protein’ through the link with 60 kDa Ro: it seems to have a role in ubiquitination and modulation of innate immune system though regulation of proinflammatory cytokines and interferon related factors. Anti-Ro antibodies recognize isolated 60 kDa Ro (which contains only conformational epitopes) or 60 kDa Ro associated with 52 kDa Ro (which has only linear epitopes). Isolated anti-52 kDa Ro antibodies can be found in SS, but they can also be frequently detected in other autoimmune disorders [51,52]. Interest has been raised for anti-52 kDa Ro, as there are new insights on the mechanisms of intracellular immunity mediated by these autoantibodies when they penetrate into cells [53].

Almost every assay can accurately detect anti-La antibodies. By contrast, the correct assays for anti-Ro antibodies should use raw or conformational antigens in order to detect autoantigens with the best sensitivity and specificity. Counterimmunoelectrophoresis, using raw spleen extracts, shows a similar performance level to the ‘gold standard’ immunoprecipitation assay. Western blotting tests, ELISAs and multiplex microbead immunoassays show variable results depending on antigen purification, synthesis and maintenance of conformational epitopes of Ro peptides [54].

Other autoantibodies can be detected in SS, as associated or substitutes of anti-Ro antibodies [46]. Anti-centromere antibodies (ACA), usually found in limited systemic sclerosis (SSc), are detected in 5% to 10% of SS cases as an alternative to anti-Ro/La antibodies. ACA-positive SS shows SSc features, such as Raynaud’s phenomenon, puffy hands, dysphagia and teleangectasia, but has a lower rate of pulmonary involvement compared to ACA + SSc. These patients show sicca symptoms not due to glandular fibrosis, as observed in SSc, but due to a high rate of lymphocyte infiltration as well as anti-Ro/La-positive SS. These data suggest that ACA-positive SS could be considered an overlap SS/SSc disease [55].

Anti-Ki/SL, anti-Ku and anti-p80 coilin antibodies are more rarely found in SS. Anti-Ki/SL antibodies, originally found in SLE with sicca, have been described in primary SS in association with anti-Ro or as isolated markers [56]. Anti-p80 coilin has been detected in SS or SSc, especially when associated with primary biliary cirrhosis [57]. Moreover, anti-Ku antibodies are usually considered markers of overlap SSc-myositis or SSc/SLE syndrome [58]. They have been detected in SS with features of SSc, cutaneous lupus and/or myositis.

In summary, most patients with SS show a mild disease with a simple autoantibody profile. Regardless, anti-Ro and La antibodies likely play a pathogenic role in inducing local inflammation and damage and are serological markers of systemic complications. Other autoantibodies, such as ACA and anti-Ku, may define a more complex disease with overlap features and different prognosis.

### Association of SS with other autoimmune diseases

SS may occur in isolation, often referred to as primary SS, or in conjunction with another connective tissue disease, most commonly RA or SLE [59]. This association is termed secondary SS, according to the AECG, even if SS was diagnosed as secondary disease many years before the primary disease [9].

SS has been described in association with a large variety of both organ-specific and systemic autoimmune diseases. In a series of 114 patients with pSS [60], a range of 13 associated autoimmune diseases was detected. In all, 38 patients (33%) were diagnosed as having 1 additional autoimmune disease, 7 (6%) had 2, and 2 (2%) had 3. The most common autoimmune disorder was hypothyroidism (14%). Similarly, a more recent cohort of 410 patients with SS [61] showed the presence of polyautoimmunity in 134 (32.6%), with thyroid disease being the most common (21.5%). The prevalence of systemic diseases such as RA and SLE was around 8%. As the presence of a concomitant autoimmune disease involves nearly one-third of patients with SS, a common pathogenic background may be advocated and the search for polyautoimmunity is warranted in every SS patient.

The relationship between SS and SLE has been recently addressed in a meta-analysis [62]. In a total of 2489 SLE patients, the estimated prevalence of SS was 17.8%. The clinical features of SLE-SS patients were found to be: (i) older age, (ii) increased frequency of oral ulcers and arthritis and (iii) proteinuria and CNS involvement, though these tended to be less frequent. With regard to autoantibodies, anti-double-stranded DNA antibodies were equally present in both groups, while anti-Ro and anti-La were more frequent, and anti-Sm and anti-cardiolipin antibodies were less prevalent in SLE-SS than SLE alone. Overall, the combined disease SLE-SS seems to be characterized by less organ involvement, a more specific autoantibody profile and a favorable clinical outcome.

The evolution towards SLE in patients with pSS has also been addressed. In a cohort of 100 patients with pSS, 15% of them could be classified as having SLE after a follow-up period of 10 years. Patients who developed SLE had a lower age, lower C3 concentration, higher level of IgG, and the presence of anti-La at the time of diagnosis of pSS [63]. In a larger cohort of 445 patients with pSS, the development of SLE was observed in only 1.3% of the patients, after a mean period of 77 months [64].

RA is frequently associated with both sicca symptoms and true sSS. In a Spanish cohort of RA patients, a cumulative prevalence of sSS was described in 17% of the patients at a disease duration of 10 years [65]. In a cohort in Austria the reported prevalence of sSS was 22% [66].

In a Greek cohort, RA patients with high titers of RF were reported to be more likely to have sSS [67]. In Finland, a doubled standardized incidence ratio for NH lymphoma in RA patients with sSS when compared with RA patients without SS was described [68].

Sicca syndrome is also common among patients with SSc due to fibrotic changes of the salivary glands. In original cohorts of SSc patients the prevalence of sSS was reported to be 17% and 29% [69,70].

In 2 more recent studies involving 133 patients with SSc and sicca syndrome (14% classified as sSS) [71] and 27 patients with SSc + sSS compared with 202 SSc patients without sSS [72], it was found that SS associated with SSc was more often complicated by peripheral neuropathy and additional autoimmune disease or autoantibodies, not typical for either pSS or SSc. It was suggested that SS may be protective against systemic sclerosis-associated pulmonary fibrosis. Limited SSc was predominantly associated with SS in these studies (81% and 95%, respectively).

There have been no studies to date of patients with mixed connective tissue disease (MCTD) that report the prevalence of SS. The development of MCTD in pSS has not been described so far [60,64]. The prevalence of anti-ribonucleoprotein autoantibodies (anti-RNP) in the absence of coexisting MCTD has been reported in 4% of patients with pSS [55].

### Novel aspects of SS: the role of infections and vitamin D

#### Infections and SS

The etiology of autoimmune diseases (AID) is multifactorial where genetic, immunologic, hormonal and environmental factors play in concert in their induction. The final step determining the date of emergence of an AID is most probably an environmental trigger, which is generally of infectious origin [73]. In the interplay between infectious agents and autoimmunity it was found that the same infectious agent (that is, Epstein-Barr Virus (EBV)) may be involved in inducing many autoimmune diseases, while the same autoimmune disease may be caused by various agents (that is, EBV, cytomegalovirus (CMV), *Helicobacter pylori*, and so on) [74].

Recently, several multicenter studies analyzed a large number of sera samples (>2,500) from patients with AID such as SS, SLE, antiphospholipid syndrome (APS), RA, vasculitides, and others for the presence of a profile of anti-infectious agents antibodies including EBV, CMV, *H. pylori*, rubella, treponema, Herpes virus and toxoplasmosis. In several diseases a higher prevalence and titers of anti-infectious antibodies were found compared with healthy controls matched for sex, age and ethnicity [75-80]. For instance, in patients with SS the prevalence and titers of antibodies against EBV-early antigen were significantly higher than in their control group (*P* = 0.0003).

Interestingly, in some diseases lower titers of anti-infectious agents were found, such as the lower prevalence and titers of rubella and CMV antibodies (IgM) detected in patients with SS compared to controls (*P* <0.02). This may allude indirectly to the notion that some infectious agents may have a protective rather than a pathogenic role for a specific autoimmune disease.

Furthermore, a certain infectious agent may determine why an individual with the ‘proper’ genetic background will develop one AID rather than others, as well as its clinical manifestations and severity.

#### Low levels of vitamin D are associated with neuropathy and lymphoma among patients with SS

The morbidity of SS is mainly determined by extraglandular disease and increased prevalence of lymphoma. Environmental and hormonal factors, such as vitamin D, may play a role in the pathogenic process and disease expression.

The levels of vitamin D and their association with manifestations of SS were studied in a large international multicenter cohort [81]. Vitamin D levels were determined in 176 patients with pSS and 163 matched healthy volunteers utilizing LIAISON chemiluminescent immunoassays (DiaSorin, Saluggia, Italy). Mean vitamin D levels were comparable between patients with SS and controls: 21.2 ± 9.4 ng/ml and 22.4 ± 10 ng/ml, respectively. Peripheral neuropathy was diagnosed in 23% of patients with SS and associated with lower vitamin D levels (18.6 ± 5.5 ng/ml vs 22.6 ± 8 ng/ml (*P* = 0.04)). Lymphoma was diagnosed in 4.3% of patients with SS, who had lower levels of vitamin D, 13.2 ± 6.25 ng/ml, compared to patients with SS without lymphoma (22 ± 8 ng/ml; *P* = 0.03). Other clinical and serological manifestations did not correlate with vitamin D status.

This study reported for the first time the presence of low vitamin D levels in patients with pSS with peripheral neuropathy. Overall, it seems that vitamin D deficiency may be a component in the pathogenesis of neuropathy in pSS, and may be used for monitoring and treatment of this condition [82-87].

Patients with pSS are at increased risk for NH lymphoma compared to healthy populations [44]. While the relationship between vitamin D and the risk for lymphoma in pSS has not been reported previously, there is some evidence from case–control studies that low dietary intake of vitamin D is associated with an increased risk for NHL in the normal population [88,89]. Vitamin D and its metabolites have been shown to have an antiproliferative effect on lymphoma cell lines and to attenuate their vitamin D receptor (VDR) expression [90].

Thus, low vitamin D levels may join low complements components and the presence of cryoglobulins in predicting eventual development of lymphoma in patients with SS.

Given the associations between hypovitaminosis D and severe complications of SS, it can be proposed that vitamin D supplementation should be given to every patient with SS.

### Sjögren’s syndrome: a female disease

Interestingly, it has also recently been claimed that vitamin D may be linked with a severe complication that may affect pregnant women with anti-Ro and anti-La: congenital heart block (CHB). CHB is the result of the passive transfer of maternal autoantibodies to the fetus in the presence of genetic predisposing factors that allow antibody-mediated cardiac damage [91].

A recent study conducted in Sweden found out that a greater proportion of children with CHB were born during the summer [92]. This means that the gestational period of enhanced CHB susceptibility (18 to 24 weeks of gestation) occurred during January to March, which is the time of the year when vitamin D levels were at their lowest. The authors concluded that the seasonal timing of the pregnancy may be critical to the onset of CHB and that vitamin D could be a possible mediator of such seasonal variation.

Aside from the severe complication of CHB, the presence of anti-Ro and anti-La antibodies does not seem to affect the gestational outcome as compared with pregnant women with autoimmune diseases negative for anti-Ro and anti-La. In a large case–control study, no difference was found in terms of pregnancy loss, intrauterine fetal deaths, preterm delivery and small-for-gestational-age infants [93]. However, when compared to age-matched healthy pregnant women, mothers with SS seem to give birth to offspring of lower birthweight and a normal delivery is less common.

Patients with SS can also suffer from gynecological problems more often than healthy women. Vaginal dryness and dyspareunia affect more than half of patients, with a significant difference with age-matched normal controls [94-96]. In addition, kissing can be difficult and unpleasant due to dry mouth [96]. These problems could lead to a relevant impairment of sexual function in women with SS [96].

The female dominance and the late onset (40 to 50 years of age) in SS can be explained by the regulatory role of sex hormones [97]. Estrogens seem to protect secretory glandular acinar cells against apoptosis while the lack of estrogens during menopause specifically leads to increased apoptosis of the exocrine cells. Conversely, the male hormone (testosterone) is converted in exocrine glands to dihydrotestosterone (DHT), which is antiapoptotic and protects against acinar cell apoptosis. Estrogen-deficient women need to produce dehydroepiandrosterone (DHEA) in the adrenal glands and convert it to DHT in the exocrine glands through complex enzymatic mechanisms. In SS, such machinery is deranged so that hormonal changes, in part systemic endocrine but predominantly local intracrine, contribute to abnormal apoptosis of secretory acinar cells. The clearance of this overload of apoptotic material may lead to the breakdown of autotolerance in immunogenetically predisposed individuals, giving rise to the complex pathogenic mechanisms of SS.

### The therapeutic challenge: old and new treatments

The therapeutic management of pSS is based on symptomatic treatment of glandular manifestations and on the use of disease-modifying drugs for systemic involvement [98]. Symptomatic treatment with saliva substitutes and eye drops is effective in the relief of sicca syndrome complaints, whereas immunomodulatory and immunosuppressive agents are used in patients with severe extraglandular manifestations and should be tailored to the specific organ involved. The aim of disease-modifying drugs is to restore the deregulated immunological pathways that are accountable for the disease process.

### Symptomatic treatment

Symptomatic treatment not only has beneficial effects on oral and ocular dryness, but can also prevent complications of sicca syndrome. In fact, untreated severe dry eye can result in corneal ulceration, vascularization, opacification and perforation, whereas dry mouth can be complicated by dental caries, oral candidiasis, and periodontal disease.

### Dry mouth topical treatment

Dry mouth topical treatment encompasses the following approaches: (a) non-pharmacological measures including adequate hydration, avoidance of irritants (coffee, alcohol, nicotine, and so on), substitution or reduction of xerostomizing drugs, meticulous oral hygiene (fluoride application, frequent dental examinations, prompt treatment of candidal infections), and sugar-free gums, lozenges and maltose lozenges to increase salivary flow; (b) saliva substitutes (mucin, caboxymethycellulose, hydroxymethilcellulose) available in the following forms: lubricating gels, mouthwashes, lozenges, toothpastes, intraoral long-release inserts and mucin spray.

A recent Cochrane review of 36 randomized controlled trials (RCTs), involving 1,597 subjects, analyzed the effect of different saliva stimulants and substitutes including lozenges, sprays, mouth rinses, gels, oils, chewing gum or toothpastes, and concluded that there is no strong evidence that any topical therapy is effective for relieving the symptoms of dry mouth [99].

The effect of saliva substitutes on patients with SS was evaluated in four RCTs, enrolling a low number of patients and using a short-term follow-up [98]. Three out of four RCTs showed an effectiveness of saliva substitutes in relieving dry symptoms, but they did not observe any increase in salivary flow.

### Dry eye topical treatment

Dry eye topical treatment approach is based on [100]: (a) non-pharmacologic measures, including avoidance of dry, smoky, windy environments, prolonged reading, computer use, use of humidifiers, goggles with side seals/moisture chambers, avoidance of aggravating drugs (diuretics, beta blockers, tricyclic antidepressants, antihistamines), and punctual occlusion in refractory cases (plugs, cauterization, surgery); (b) replacement of tear volume, that is, artificial tears (preservative-free products, hypotonic solutions, and emulsions), autologous serum eye drops and platelet releasate, which are promising treatments especially for patients intolerant to artificial tears or with refractory KCS (the major limitation to a widespread use of these products is related to their preparation and preservation); (c) topical drugs counting ciclosporin A, which was approved for the treatment of dry eye by the US Food and Drug administration (FDA) but not by the European Medicine Agency (EMA), corticosteroids, and non-steroidal anti-inflammatory drugs (NSAIDs).

There are few rigorous studies on the effect of topical medications for eyes in patients with SS. As far as artificial teardrops are concerned, emulsions containing hyaluronate and hydroxypropylmethyl-cellulose, hypotonic solutions that decrease the tear film osmolality, and preservative-free products that are less irritating when applied chronically on a daily basis, seem to be the best options [98].

In patients with severe KCS, topical NSAIDs can be effective in relieving ocular pain, but they should only be used for a short time and under medical supervision since they reduce corneal sensitivity, predisposing users to corneal damage.

Patients with severe dryness and refractory KCS may also require topical corticosteroid treatment. Although glucocorticoids exert a rapid and intense anti-inflammatory effect, they should only be used for a short time since they can induce severe side effects such as glaucoma and cataracts.

A number of studies were carried out with the use of topical ciclosporin A in patients with KCS and SS, showing good results in terms of dry symptom relief and tear production.

### Systemic drugs for sicca symptoms

Secretagogues are indicated in patients with moderate or severe SS who have dryness and residual esocrinal gland function [100]. Muscarinic receptor agonists, that is, pilocarpine and cevimeline, have been used for both dry mouth and dry eye and data from RCTs demonstrated a substantial benefit on sicca symptoms, and improvements in salivary flow rate and ocular tests results. Cevimeline was approved for the treatment of dry mouth and dry eye by the FDA but not by the EMA. The most frequent side effects of muscarinic receptor agonist therapy are sweating, increased urinary frequency, and flushing. Mucolytic agents, that is bromhexine or *N*-acetylcysteine, have been used for dry mouth although evidence of their efficacy is lacking.

### Disease-modifying drugs

All the drugs currently used in the treatment of autoimmune rheumatic diseases have also been administered to patients with pSS in order to improve sicca symptoms and modify the immune inflammatory pathways involved in disease progression [98]. Unfortunately, evidence supporting the use of these agents is limited.

### Corticosteroids

There are too few studies on oral corticosteroid treatment in patients with SS to draw definitive conclusions. Corticosteroids at high dosage downregulate the immune inflammatory process within the salivary and lacrimal glands [101], but there is no evidence that they increase salivary and lacrimal flow rates. In addition, the chronic use of corticosteroids at high dosage should be avoided in order to prevent severe side effects. Thus, corticosteroids are currently used primarily in patients with extraglandular manifestations or in cases with parotid swelling.

### Antimalarials

Antimalarial agents have been shown to improve sicca features and constitutional symptoms such as fatigue and arthromyalgia [102,103]. Moreover, hydroxychloroquine has been reported to increase salivary flow rate by inhibiting glandular cholinesterase [104], decrease inflammatory indices, that is, ESR and C reactive protein (CRP), and immunological abnormalities, that is, γ-globulin, IgG, IgM, RF, anti-Ro, anti-La. Notably, a decrease of B cell activating factor (BAFF) in the tear fluid of patients using hydroxychloroquine has recently been reported [103].

Importantly, hydroxychloroquine has recently been shown to exhibit antineoplastic properties. In fact, it seems to prevent mutations in cells with high mitotic rate as well as to increase cellular mechanisms of DNA protection and repair [105]. This is an interesting finding since patients with pSS have a significantly higher risk of developing lymphoma than the general population.

### Immunosuppressants

Immunosuppressant agents as ciclosporin A, azathioprine, methotrexate, mycophenolic acid and leflunomide are all used empirically in SS. Indeed, only a few studies including a low number of patients and using a short-term follow-up (6 months) have been published; therefore, their conclusions have a low level of evidence. A benefit on sicca symptoms without significant improvement in objective tests has been reported by some of them. These drugs are currently used in the treatment of extraglandular manifestations and tailored to the organ specific involvement [106].

### Biological drugs

No biologic drugs are currently approved for pSS. However, some published studies have analyzed the off-label therapeutic potential of the following biological agents in pSS: tumor necrosis factor (TNF)α antagonists (etanercept and infliximab), anti-CD20 and anti-CD22 monoclonal antibodies (mAbs).

After three open-label studies in which anti-TNFα agents were shown to improve glandular and extraglandular manifestations, two RCTs failed to demonstrate the superiority of infliximab and etanercept over placebo [107]. Since then, no further studies with the use of these agents have been carried out. Notably, increased type I interferon (IFN)-pathway activation and elevated BAFF serum levels in patients with SS treated with etanercept have been shown [108]. Since type I IFN and BAFF seem to be involved in the pathogenesis of SS as well as of other autoimmune diseases [42], anti-TNFα agents should be avoided in patients with autoimmune diseases, including SS.

A number of uncontrolled studies and two RCTs have been published on anti-CD20 treatment (rituximab) in patients with SS (Table 2) [109-120]. In uncontrolled studies, rituximab was found to be effective in controlling extraglandular manifestations of the disease including arthritis, skin vasculitis, particularly when associated with cryoglobulins, fatigue, and quality of life; however, only a modest effect on sicca features was demonstrated [121].

**Table 2:** Studies including patients affected with Sjögren’s syndrome (SS) treated with rituximab

**Author, year and reference**	**No. of patients**	**Study design**	**Involvement**	**Efficacy**	**Safety**
Somer *et al*., 2003 [109]	1	Case report	Marginal zone lymphoma, xerophthalmia, xerostomia	Improvement in corneal staining, Schirmer’s test, salivary flow rate, tear production, salivary pooling, diminished parotid enlargement	No AE reported
Voulgarelis *et al*., 2004 [110]	4	Case reports	Marginal zone lymphoma, parotid gland enlargement, lymphadenopathy, cryoglobulinemia, purpura, peripheral neuropathy, arthralgia	Improvement in lymphoma, arthralgia, cryoglobulinemia, purpura, peripheral neuropathy (50%)	No AE reported
Gottenberg *et al*., 2005 [111]	6	Retrospective	2 MALT lymphomas, 2 vasculitis with cryoglobulinemia, 2 parotid gland enlargement and articular involvement	Improvement in parotid swelling, subjective dryness, fatigue and vasculitis in 5 out of 6 patients	1 serum sickness, 1 infusion reaction
Pijpe *et al*., 2005 [112]	15	Open label	8 early primary SS and 7 MALT lymphoma: 8 parotid gland swelling, 8 Raynaud’s phenomenon, 13 fatigue, 11 arthralgia, 2 pulmonary involvement, 2 vasculitis	Remission of lymphoma in 3 of 7 patients, disease stability in 3 of 7, progression in 1. Increased salivary secretion, improvement in the rose Bengal score and tear, BUT, mouth dryness, arthralgia. No improvement on Schirmer test.	2 infusion reactions, 1 Herpes zoster
Ring *et al*., 2006 [113]	1	Case report	Distal renal tubular acidosis, xerostomia with mouth ulcerations	Xerostomia improvement	No AE reported
Seror *et al*., 2007 [114]	16	Retrospective	5 lymphoma, 2 pulmonary involvement, 2 polysynovitis, 5 mixed cryoglobulinemia, 1 thrombocytopenia, and 1 mononeuritis multiplex	Remission of lymphoma in 4 of 5 patients; improvement of systemic involvements in 9 of 11, subjective dryness in 5 of 16, and regression of keratitis in 2 of 11. No response on salivary flow and Schirmer test.	2 infusion reactions, 1 serum sickness
Devauchelle-Pensec *et al*., 2007 [115]	16	Open label	Sicca symptoms, pain, fatigue; 1 pulmonary involvement	Improvement in subjective fatigue, pain, dryness, and pulmonary involvement. No changes in unstimulated salivary flow, salivary gland score and ophthalmologist evaluation.	3 infusion reactions, 1 serum sickness
Dass *et al*., 2008 [116]	8 (PL 9)	RCT	Fatigue, ocular and mouth dryness; no systemic involvement	Improvement in fatigue, general health and SF-36. No improvement on Schirmer test and salivary flow rate.	2 infusion reactions, 1 delayed reaction with meningism, 1 gastroenteritis and palpitation
Galarza *et al*., 2008 [117]	8	Open label	Severe glandular and musculoskeletal involvement, cutaneous vasculitis	Improvement in parotid swelling, articular involvement, fatigue, and subjective dryness in 4 of 7 patients	3 AE: 2 infusion reactions
Ramos-Casals *et al*., 2010 [118]	15	Registry	6 lymphoma, 4 neurological involvement, 2 hematological involvement, 1 refractory glomerulonephritis, arthritis, and protein-losing enteropathy	Complete response in 67% of patients, partial response in 20%, no response in 13%	1 urinary tract infection, 1 interstitial pneumonitis
Meijer *et al*., 2010 [119]	20 (PL 10)	RCT	15 arthralgia, 6 arthritis, 2 renal involvement, 1 peripheral neuropathy, 11 Raynaud’s phenomenon, 17 tendomyalgia, 6 vasculitis, thyroid dysfunction	Improvement in saliva flow rate, stimulated lacrimal gland function; but not in BUT and Schirmer test. Improvement in SF-36 and MFI. Improvement in extraglandular manifestations.	1 serum sickness, 12 infections in 11 patients on rituximab vs 7 infections in 4 patients on placebo
Mekinian *et al*., 2012 [120]	17	Registry	All peripheral nervous system involvement: 10 patients with cryoglobulinemia and/or vasculitis, 7 patients without cryoglobulinemia and/or vasculitis	Response in 9 of 10 patients with cryoglobulinemia and/or vasculitis, and in 2 of 7 without cryoglobulinemia and/or vasculitis	6 (35%) AE: 2 mild arterial hypertension, 1 infusion reaction, 1 cutaneous infection, CMV infection, hypogammaglobulinemia

AE, adverse event; BUT, break-up time; CMV, cytomegalovirus; MALT, mucose-associated lymphoid tissue; MFI, multidimensional fatigue inventory; PL, placebo; RCT, randomized controlled trial; SF-36, Short Form 36 Health Survey.

In a recently published RCT, 20 patients affected with active primary SS and residual salivary gland function were treated with rituximab and compared to 10 patients on placebo [119]. In comparison with baseline values, rituximab treatment significantly improved the stimulated whole saliva flow rate and several other variables including B cell number, RF levels, unstimulated whole saliva flow rate, lacrimal gland function, fatigue, quality of life, and sicca symptoms. Interestingly, the drug effect lasted 24 weeks and stimulated whole saliva flow rate declined when CD20+ B cells started to repopulate. Despite these promising results, it has been recently shown that rituximab treatment does not alter the characteristic features of increased clonal expansions seen in the parotid salivary glands of patients with pSS [122]. The presence of clonally related immunoglobulin producing cells before and after rituximab treatment strongly suggests that immunoglobulin-producing cells persist in the salivary glands of patients with pSS despite B cell depletion, which can account for disease relapse after treatment [122].

Anti-CD22 mAb (4 infusions of 360 mg/m^2^ of epratuzumab once every 2 weeks) was administered to 16 patients with SS in an open-label, phase I/II study, with 6 months of follow-up [123]. A substantial number of patients achieved a significant clinical response based on a composite endpoint and the drug was well tolerated. Epratuzumab acts through a downregulation of CD22, which is overexpressed in the peripheral B cells of patients with SS. According to these preliminary findings, epratuzumab seems to be a promising treatment in patients with SS.

## Conclusions

SS is rather far from being considered a simple disease of ‘dry mouth and dry eyes’. Research on SS is extremely active and aims at improving the classification of patients through more objective criteria (for example, the 2012 SICCA Criteria), probing deeper into the etiology and the complex pathogenesis of the disease and providing evidence for the use of new targeted treatments, such as anti-B cell drugs. The role of infections in the emergence of SS has been recently addressed, showing that some infectious agents may promote the disease, while others may have a protective action against the development of autoimmunity. Extraglandular manifestations are still a challenge in the management of SS, among which the most serious is B cell NH lymphoma. The recent finding that severe complications such as lymphoma and peripheral neuropathy are associated with low vitamin D levels opens new avenues in the understanding of the disease and in its treatment. The fact that CHB is also more frequent during winter and associates with hypovitaminosis D supports the idea that the role of vitamin D should be further investigated in SS and adequate supplementation should be given to these patients.

## Abbreviations

ACA: Anti-centromere antibodies; AECG: American-European Consensus Group; AID: Autoimmune diseases; ANA: Anti-nuclear antibodies; Anti-La: Anti-La/SS-B antibodies; Anti-Ro: Anti-Ro/SS-A antibodies; APS: Antiphospholipid syndrome; BAFF: B cell activating factor; CHB: Congenital heart block; CMV: Cytomegalovirus; CRP: C-reactive protein; DEWS: Dry Eye Workshop; DHT: Dihydrotestosterone; EBV: Epstein-Barr virus; EMA: European Medicine Agency; ESR: Erythrocyte sedimentation rate; FDA: Food and Drug Administration; GMN: Glomerulonephritis; IFN: Interferon; ILD: Interstitial lung disease; IN: Interstitial nephritis; KCS: Keratoconjuctivitis sicca; LFU: Lacrimal functional unit; LIP: Lymphocytic interstitial pneumonia; MALT: Mucosa-associated lymphoid tissue; MCTD: Mixed connective tissue disease; NH: Non-Hodgkin’s; NSAIDs: Non-steroidal anti-inflammatory drugs; NSIP: Non-specific interstitial pneumonia; PNS: Peripheral nervous system; RCT: Randomized controlled trial; RF: Rheumatoid factor; SICCA: Sjögren’s International Collaborative Clinical Alliance; SS: Sjögren’s syndrome; SSc: Systemic sclerosis; pSS: Primary Sjögren’s syndrome; sSS: Secondary Sjögren’s syndrome; UIP: Usual interstitial pneumonia; US: Ultrasound; VDR: Vitamin D receptor.

## Competing interests

The authors declare that they have no competing interests.

## Authors’ contributions

All authors were involved in drafting the manuscript and approved the final version.

## Pre-publication history

The pre-publication history for this paper can be accessed here:

http://www.biomedcentral.com/1741-7015/11/93/prepub
